# Genetic analyses in a cohort of 191 pulmonary arterial hypertension patients

**DOI:** 10.1186/s12931-018-0789-9

**Published:** 2018-05-09

**Authors:** Hang Yang, Qixian Zeng, Yanyun Ma, Bingyang Liu, Qianlong Chen, Wenke Li, Changming Xiong, Zhou Zhou

**Affiliations:** 1 State Key Laboratory of Cardiovascular Disease, Beijing Key Laboratory for Molecular Diagnostics of Cardiovascular Diseases, Diagnostic Laboratory Service, Fuwai Hospital, National Center for Cardiovascular Diseases, Chinese Academy of Medical Sciences and Peking Union Medical College, Beijing, China; 2 State Key Laboratory of Cardiovascular Disease, Center of Pulmonary Vascular Disease, Fuwai Hospital, National Center for Cardiovascular Diseases, Chinese Academy of Medical Sciences and Peking Union Medical College, Beijing, China; 3 Fuwai Hospital, No.167, Beilishi Road, Xicheng District, 100037 Beijing, People’s Republic of China

**Keywords:** Pulmonary arterial hypertension, Genetic analyses, Genotype-phenotype correlation

## Abstract

**Background:**

Pulmonary arterial hypertension (PAH) is a progressive and fatal disorder associated with high pulmonary artery pressure. Genetic testing enables early diagnosis and offers an opportunity for family screening. To identify genetic mutations and help make a precise diagnosis, we performed genetic testing in 191 probands with PAH and tried to analyze the genotype-phenotype correlation.

**Methods:**

Initially, PAH samples (*n* = 119) were submitted to *BMPR2* screening using Sanger sequencing. Later, we developed a PAH panel test to identify causal mutations in 13 genes related to PAH and tried to call *BMPR2* copy number variations (CNVs) with the panel data. Multiplex ligation-dependent probe amplification (MLPA) was used to search for CNVs in *BMPR2*, *ACVRL1* and *ENG*. Notably, *EIF2AK4* gene was also involved in the panel, which allowed to distinguish pulmonary veno-occlusive disease (PVOD)/pulmonary capillary hemangiomatosis (PCH) patients from idiopathic PAH (IPAH). Characteristics of patients were compared using t test for continuous variables.

**Results:**

Pathogenic *BMPR2* mutations were detected most frequently in 32 (17.9%) IPAH and 5 (41.7%) heritable PAH (HPAH) patients by sequencing, and 12 *BMPR2* CNVs called from the panel data were all successfully confirmed by MLPA analysis. In addition, homozygous or compound heterozygous *EIF2AK4* mutations were identified in 6 patients, who should be corrected to a diagnosis of PVOD/PCH. Genotype-phenotype correlation analysis revealed that PAH patients with *BMPR2* mutations were younger at diagnosis (27.2y vs. 31.6y, *p* = 0.0003) and exhibited more severe pulmonary hemodynamic impairment and a worse cardiac index compared with those without *BMPR2* mutations.

**Conclusions:**

The panel assay represented a highly valuable tool in PAH genetic testing, not only for the detection of small sequence alterations, but also for an indication of *BMPR2* CNVs, which had implications for the specific samples to perform further MLPA assay. Analyses of PAH causal genes have a great help to clinical diagnosis and deep implications in disease treatment.

**Electronic supplementary material:**

The online version of this article (10.1186/s12931-018-0789-9) contains supplementary material, which is available to authorized users.

## Background

Pulmonary arterial hypertension (PAH) is a progressive and fatal disorder, which is diagnosed by a mean pulmonary artery pressure ≥ 25 mmHg at rest accompanied by a pulmonary artery wedge pressure (PAWP) ≤ 15 mmHg and a pulmonary vessel resistance (PVR) > 3 Wood units (WU), excluding other known causes of pulmonary hypertension, e.g., pulmonary diseases, thromboembolic diseases and left heart diseases [1]. Given the nonspecific clinical manifestations in the early stage, patients with PAH are frequently diagnosed late. Whenever the pulmonary pathologic changes become advanced and irreversible, prognosis and survival are poor and pessimistic. It has been reported that more than 20% patients have a two-year delay in the diagnosis of PAH [2], and if not treated timely, patients might progress into right heart failure [3] and the average survival duration after diagnosis is 2.8 years [4]. Idiopathic PAH (IPAH) corresponds to sporadic disease, without any familial history of PAH or known causes, which has an estimated prevalence of 5.9/1,000,000. Heritable PAH (HPAH) is diagnosed when there is a positive family history or when a pathogenic variant has been identified. For individuals with PAH, the most effective therapy to date is continuous injection of prostacyclin analogs. Other options include calcium channel blockers, oral prostanoids, phosphodiesterase type 5 inhibitors, and endothelin receptor antagonists [5]. Lung transplantation is effective, but long-term post-surgical survival is limited [6].

Since the 1990s, much has been learned about the molecular and genetic factors related to PAH. *BMPR2* mutation is identified as the main genetic cause of PAH, accounting for 75%–90% of familial PAH [7, 8] and 3.5%–40% of sporadic cases [7, 9], with an autosomal dominant inheritance pattern [10]. The *BMPR2* gene encodes bone morphogenetic protein receptor type II, which belongs to the transforming growth factor-β (TGF-β) superfamily and is involved in the regulation of cell growth and apoptosis. Two other genes, *ACVRL1* [11] and *ENG* [12], which also encode receptors belonging to the TGF-β superfamily, have been more recently identified in PAH accompanying hereditary hemorrhagic telangiectasias (HHT). Similarly to *BMPR2*, these two genes affect vascular proliferation. With the development of genetics, more genes have been discovered to be implicated in the pathogenesis of PAH, including *KCNK3* [13]*, CAV1* [14], *SMAD9* [15], *BMPR1B* [16], but considerably less commonly so (1%–3%). The discovery of a causative mutation could help make an early diagnosis and provide an opportunity for family screening [17].

Pulmonary veno-occlusive disease (PVOD) is a rare disease that shares several clinical and hemodynamic similarities with IPAH but is distinct from IPAH in pathology and prognosis. An accurate diagnosis of PVOD in the early stage is crucial for suitable drug therapy and timely option for lung transplantation. Histological examination of lung biopsy is the gold standard for the diagnosis of PVOD, but it is often too invasive and unacceptable for the patient. Recent discoveries have indicated that gene mutations in eukaryotic translation initiation factor 2 alpha kinase 4 (*EIF2AK4*) are responsible for inherited PVOD and pulmonary capillary hemangiomatosis (PCH) [18], and the 2015 ESC/ERS Guidelines declared that identification of a biallelic *EIF2AK4* mutation was sufficient to make a diagnosis of PVOD/PCH without histological confirmation [1].

To identify the causal mutations in PAH patients and differentiate PVOD patients from those with IPAH, a custom gene panel targeting PAH and related diseases was used to test 191 probands with clinically suspected IPAH and HPAH. Furthermore, the performance of calling copy number variations (CNVs) from our panel data was evaluated.

## Methods

### Subjects

One hundred and ninety-one PAH patients referred by the Center of Pulmonary Vascular Disease at Fuwai Hospital between 2016 and 2017 were enrolled in our study. All patients underwent a detailed clinical examination, and other known causes of pulmonary hypertension were excluded by an expert physician.

### Sanger sequencing

Genomic DNA was extracted from EDTA–anticoagulated whole blood using a Wizard® Genomic Purification System A1125 (Promega, USA) kit according to the manufacturer’s recommended protocol. All 13 exons in the *BMPR2* coding sequence region and a minimum of 20 base pairs of intronic DNA flanking each exon were amplified by PCR (Tiangen Biotech, Beijing, China). Briefly, the PCR program consisted of initial denaturation for 10 min at 95 °C; followed by 30 cycles of 30 s at 95 °C, 30 s at 55 °C and 30 s at 72 °C; and a final extension at 72 °C for 7 min. Then, the PCR products were purified with a TIANgel Midi Purification Kit (Tiangen Biotech, Beijing, China) and subsequently sequenced using a BigDye Terminator Cycle sequencing kit V 3.1 (ABI Biosystems) on an ABI PRISM 3730 Sequence Analyzer according to the manufacturer’s directions.

### Gene panel testing

A total of 13 genes associated with heritable PAH and related diseases, such as HHT and PVOD/ PCH were involved in our panel. Referring to GeneReview, HGMD, OMIM and literatures, well established causative genes (PAH: *BMPR2* (bone morphogenetic protein receptor type 2) [19]*, KCNK3* (potassium two pore domain channel subfamily K member 3) [13]*, CAV1* (caveolin 1) [14]*, SMAD9* (SMAD family member 9) [15], *BMPR1B* (bone morphogenetic protein receptor type 1B) [16]; HHT: *ACVRL1* (activin A receptor like type 1) [11]*, ENG* (endoglin) [12]*, GDF2* (growth differentiation factor 2) [20]*, SMAD4* (SMAD family member 4) [21]; PVOD/PCH: *EIF2AK4* (eukaryotic translation initiation factor 2 alpha kinase 4) [18]) and another three strong candidate genes with supportive functional studies, *KCNA5* (potassium voltage-gated channel subfamily A member 5) [22], *NOTCH3* (notch 3) [23], and *TOPBP1* (DNA topoisomerase II binding protein 1) [24], were included. A custom-designed gene panel (Roche NimbleGen, USA) was used for gene testing. The size of the panel was 87 kb, with a coverage of 98.7% of the target regions.

Library preparation was performed according to the manufacturer’s instructions (NEBNext Ultra DNA Library Prep Kit, Illumina, USA). Briefly, 1 μg of genomic DNA was sheared to 200 bp fragments, ligated with adaptors and size-selected for PCR amplification. Every 4 amplified DNA samples were pooled and captured using the customized gene panel with biotinylated oligos (SeqCap EZ Choice Library, Roche NimbleGen, USA). The enrichment libraries were sequenced by an Illumina MiSeq 2000 sequencer (Illumina, San Diego, CA) using 2 × 150 paired-end sequencing (MiSeq Reagent Kit v2, Illumina, San Diego, CA).

### Bioinformatics analysis

Only high-quality reads were retrieved by filtering out low-quality reads and adaptor sequences using Trimmomatic software. The clean read sequences were aligned to the human reference genome (hg19) by Burrow-Wheeler Aligner (BWA), and PCR duplicates were marked by the Picard software. SNPs and insertions/deletions were identified using the GATK HaplotypeCaller program (http://www.broadinstitute.org/gsa/wiki/index.php/Home_Page) and further annotated with comprehensive ANNOVAR software for their frequencies in the Genome Aggregation Database (gnomAD) for the pathogenicity and splicing-altering prediction of single nucleotide variants in the dbNSFP database, which included results from SIFT, Ployphen-2, MutationTaster, the dbscSNV database. In addition, the clinical significance of the sequences was annotated using Clinvar (http://www.ncbi.nlm.nih.gov/clinvar/), OMIM (http://omim.org/), Uniprot (http://www.uniprot.org/) and HGMD (http://www.hgmd.org). Variants with cutoff values greater than 0.6 in the dbscSNV database were defined as splice-altering. Other synonymous variants that did not fulfill the above mentioned conditions were removed. Considering that most genes related to PAH were autosomal-dominant inherited, variants with a minor allele frequency (MAF) of > 0.1% in panel genes other than *EIF2AK4* were excluded from further analysis as polymorphism variants, while the variants in *EIF2AK4* with a MAF of > 1% were excluded because of an autosomal-recessive inherited pattern.

### Variant classification

Variants were analyzed for pathogenicity according to the recommendations of the American College of Medical Genetics (ACMG). Specifically, the analysis was based on the following criteria: (i) whether they were previously reported by a functional study or family segregation study; (ii) the nature of the variant (e.g., nonsense, frameshift indel, or splicing mutations (intron ±1 or ± 2)); (iii) variant frequency in population databases; (iv) conservation of the altered residue; (v) in silico prediction (SIFT, PholyPhen2, or MutationTaster); (vi) de novo mutation; and (vii) family segregation studies. Based on this information, a variant was classified into one of the 5 following categories: benign, likely benign, unknown significance, likely pathogenic or pathogenic.

### Copy-number variation calling

All CNVs were called by panelcn.MOPS [25], which was designed to detect targeted next-generation-sequencing (NGS) panel data. Sequencing quality was checked, and the mean coverage of sequencing was 98.7%. Duplications were removed by the Picard software before CNV calling. Samples with a high correlation of read counts (RCs) were selected automatically as controls from all the samples by panelcn.MOPS. Other parameters were kept default.

### Multiplex ligation-dependent probe amplification (MLPA)

To detect the CNVs in *BMPR2*, the commercially available MLPA kit P093-C2 was used, containing probes for *BMPR2*, *ENG* and *ACVRL1* (MRC-Holland, Amsterdam, The Netherlands). MLPA was performed according to the manufacturer’s protocol (www.mlpa.com), and the generated fragments were detected using an ABI 3500XL DX capillary electrophoresis system (Applied Biosystems). The results were analyzed with the Coffalyser software (MRC-Holland).

### Statistical analysis

Unpaired t-test was used to analyze the difference of continuous variables between different genotypes by GraphPad Prism 5. The data were presented as mean ± standard deviation (SD) and *p* < 0.05 was considered statistically significant. All statistical tests were two-sided.

## Results

A total of 191 patients were enrolled in our study and underwent genetic testing. Of the patients, 179 suffered from IPAH and 12 had HPAH. The main clinical findings are summarized in Table 1.

**Table 1:** Characteristics of patients with IPAH and HPAH who underwent a genetic testing

Characteristic	IPAH (*n* = 179)	HPAH (*n* = 12)
Age at diagnosis, y	30.7 ± 10.6	24.8 ± 7.2
Female sex	135 (75.4%)	7 (58.3%)
NYHA I-II	82 (45.8%)	6 (50.0%)
NYHA III	91 (50.8%)	5 (41.7%)
NYHA IV	6 (3.4%)	1 (8.3%)
Hemodynamics at diagnosis		
RAP, mmHg	5.2 ± 4.2	2.8 ± 4.5
mPAP, mmHg	56.1 ± 14.7	61.6 ± 16.4
PVR, dyn*s*cm^− 5^	1155.6 ± 523.6	1052.8 ± 422.4
PAWP, mmHg	7.1 ± 3.5	7.1 ± 3.1
CI, L/min/m^2^	2.8 ± 0.9	3.1 ± 1.2
SvO_2_, %	67.8 ± 7.5	71.4 ± 6.3
Peak VO_2_, ml/min/Kg	13.1 ± 3.6	12.2 ± 2.2
6-min walk distance, m	415.6 ± 101.3	435.5 ± 100.3
NT-proBNP, pg/ml	1403.6 ± 1327.4	970.1 ± 881.8

Values are mean ± SD or n (%). NYHA, New York Heart Association functional class; *RAP* right atrial pressure, *mPAP* mean pulmonary artery pressure, *PVR* pulmonary vascular resistance, *PAWP* pulmonary artery wedge pressure, *CI* cardiac index, *SvO2* mixed venous oxygen saturation, *Peak VO2* peak oxygen consumption, *NT-proBNP* N-terminal pro–B-type natriuretic peptide

All samples were completed to perform high-quality genetic testing. Initially, a total of 119 PAH samples were submitted to single-gene testing of *BMPR2* by Sanger sequencing. Later, we developed a PAH panel test in our lab. Then 97 samples with negative results in the *BMPR2* test and 72 subsequently referred samples were then directed to a panel test involving 13 genes related to PAH. If the samples were still negative, MLPA testing was continued to search for the causative mutation (Additional file 1: Figure S1).

Sequencing of the 13 PAH genes (Additional file 1: Table S1) in the 169 probands yielded a mean depth of ~ 350× and coverage of 98.7%. Exons with low (< 20×) or no coverage were subjected to Sanger sequencing to obtain 100% coverage in the main gene, *BMPR2*. In addition, potential pathogenic mutations and rare variants of unknown significance (VUS) were confirmed using Sanger sequencing.

Causal mutations identified in the sequencing tests (both *BMPR2* sequencing and panel sequencing), except for biallelic *EIF2AK4*, are described in Table 2. The table indicates that *BMPR2* mutations were detected most frequently in 32 (17.9%) IPAH and 5 (41.7%) HPAH patients, while VUS are summarized in Additional file 1: Table S2. Notably, 4 HPAH families in our cohort indicated no suspected mutations either by panel sequencing or MLPA detection (Additional file 1: Figure S2), which required further genetic testing for the identification of new genes potentially related to PAH. In addition, in 6 patients clinically diagnosed with IPAH, homozygous or compound heterozygous *EIF2AK4* mutations were detected (Table 3); thus, their diagnosis had to be corrected to PVOD/PCH.

**Table 2:** Pathogenic and likely pathogenic mutations in PAH-related genes except for *EIF2AK4*

Patient	Gene	Transcript	Exon/Intron	Nucleotide change	Protein change	De novo	Pathogenicity	Ref PMID	MAF
PAH1	*BMPR2*	NM_001204	exon5	c.551dupA	p.His184fs	NA	P	NA	NA
PAH10	*BMPR2*	NM_001204	exon6	c.793_796del	p.Glu265fs	NA	P	NA	NA
PAH17	*BMPR2*	NM_001204	exon6	c.631C > T	p.Arg211Term	NA	P	NA	NA
PAH25	*ACVRL1*	NM_001077401	exon6	c.994A > T	p.Lys332Term	NA	P	NA	NA
PAH27	*BMPR2*	NM_001204	exon3	c.338dupA	p.Tyr113_Arg114delinsTerm	NA	P	NA	NA
PAH31	*BMPR2*	NM_001204	exon4	c.439C > T	p.Arg147Term	NA	P	11,115,378	8.96 × 10^−6^
PAH34	*BMPR2*	NM_001204	exon6	c.705delA	p.Asn236fs	NA	P	NA	NA
PAH39	*NOTCH3*	NM_000435	exon11	c.1493–2- > C		NA	P	NA	9.78 × 10^−5^
PAH42	*BMPR2*	NM_001204	exon7	c.893delG	p.Val299Term	NA	P	NA	NA
PAH43	*BMPR2*	NM_001204	exon1	c.71_76del	p.24_26del	NA	LP	NA	NA
PAH46	*BMPR2*	NM_001204	exon6	c.631C > T	p.Arg211Term	De novo	P	11,115,378	NA
PAH47	*BMPR2*	NM_001204	exon12	c.2567delC	p.Thr856fs	NA	P	NA	NA
PAH51	*BMPR2*	NM_001204	exon10	c.1372C > T	p.Gln458Term	NA	P	21,737,554	NA
PAH67	*BMPR2*	NM_001204	exon7	c.944 T > C	p.Leu315P	De novo	LP	NA	NA
PAH75	*BMPR2*	NM_001204	exon8	c.1089delG	p.Val364fs	Inherited	P	NA	NA
PAH76	*BMPR2*	NM_001204	exon1	c.47G > A	p.Trp16Term	NA	P	15,687,131	NA
PAH86	*ACVRL1*	NM_001077401	exon9	c.1450C > T	p.Arg484Trp	De novo	P	11,484,689	NA
PAH95	*ENG*	NM_000118	exon3	c.247C > T	p.Gln83Term	NA	P	15,517,393	NA
PAH98	*BMPR2*	NM_001204	exon6	c.631C > T	p.Arg211Term	NA	P	11,015,450	NA
PAH102	*BMPR2*	NM_001204	exon12	c.1730 T > A	p.Leu577Term	NA	LP	NA	NA
PAH110	*BMPR2*	NM_001204	exon11	c.1471C > T	p.Arg491Trp	NA	LP	NA	NA
PAH117	*BMPR2*	NM_001204	exon2	c.178 T > C	p.Cys60Arg	NA	LP	NA	NA
PAH123	*BMPR2*	NM_001204	intron3	c.418 + 4A > G		NA	LP	NA	NA
PAH137	*ACVRL1*	NM_001077401	exon9	c.1450C > T	p.Arg484Trp	NA	P	11,484,689	NA
PAH138	*BMPR2*	NM_001204	exon9	c.1249insAT	p.Phe417fs	NA	P	NA	NA
PAH143	*BMPR2*	NM_001204	exon12	c.1789C > T	p.Arg597Term	NA	P	NA	NA
PAH150	*BMPR2*	NM_001204	exon8	c.994C > T	p.Arg332Term	NA	P	11,015,450	NA
PAH153	*BMPR2*	NM_001204	exon12	c.2617C > T	p.Arg873Term	NA	P	10,903,931	NA
PAH155	*BMPR2*	NM_001204	exon12	c.1789C > T	p.Arg597Term	NA	P	NA	NA
PAH159	*BMPR2*	NM_001204	exon12	c.2470C > T	p.Gln824Term	Inherited	LP	NA	NA
PAH163	*BMPR2*	NM_001204	exon12	c.2522 delA	p.His841fs	NA	P	18,356,561	NA
PAH166	*BMPR2*	NM_001204	exon12	c.2710_2831dup	p.Ala945fs	NA	P	NA	NA
PAH168	*ENG*	NM_000118	exon12	c.1503_1504insG	p.Gly501fs	NA	P	NA	NA
PAH176	*BMPR2*	NM_001204	exon6	c.631C > T	p.Arg211Term	NA	P	11,015,450	NA
PAH177	*BMPR2*	NM_001204	exon6	c.631C > T	p.Arg211Term	NA	P	11,015,450	NA
PAH178	*BMPR2*	NM_001204	exon6	c.713_714del	p.238_238del	Inherited	LP	NA	NA
PAH182	*ACVRL1*	NM_001077401	exon7	c.1232G > A	p.Arg411Gln	Inherited	LP	8,640,225	6.55 × 10^−5^
PAH183	*BMPR2*	NM_001204	intron6	IVS6 + 3A > C		NA	LP	NA	NA
PAH184	*BMPR2*	NM_001204	exon4	c.439C > T	p.Arg147Term	NA	LP	11,115,378	8.96 × 10^−6^
PAH187	*BMPR2*	NM_001204	exon8	c.1089delG	p.Leu363fs	NA	P	NA	NA
PAH188	*ACVRL1*	NM_001077401	exon7	c.1121G > A	p.Arg374Gln	NA	LP	12,700,602	4.49 × 10^− 5^
PAH189	*ACVRL1*	NM_001077401	exon3	c.372delC	p.Gly124fs	De novo	P	NA	NA
PAH192	*BMPR2*	NM_001204	exon12	c.2268_2269insA	p.Thr756fs	NA	P	NA	NA
PAH194	*BMPR2*	NM_001204	exon9	c.1243G > A	p.Glu415Lys	NA	LP	NA	NA
PAH195	*BMPR2*	NM_001204	exon9	c.1161delG	p.Val387fs	De novo	P	NA	NA
PAH207	*BMPR2*	NM_001204	exon7	c.901 T > C	p.Ser301Pro	De novo	LP	16,429,395	NA

*fs* frame shift, *NA* not available, *LP* likely pathogenic, *P* pathogenic, *ref. PMID* reference PubMed unique identifier, *MAF* minor allele frequency in the Genome Aggregation Database (gnomAD)

**Table 3:** Pathogenic biallelic *EIF2AK4* mutations

Patient ID	Sex/Age	Initial Diagnosis	Variant (1)	Variant (2)	Final Diagnosis
PAH3	F/21	IPAH	c.2403 + 1G > A, het	c.2632-1G > A, het	PVOD
PAH48	F/39	IPAH	c.2965C > T: p.Arg989Trp, het	c.4724 T > C: p.Leu1575Pro, het	PVOD
PAH151	M/26	IPAH	c.4414_4417del: p.1472_1473del, hom		PVOD
PAH169	F/51	IPAH	c.168delT: p.Asn56fs, het	c.4660-1G > A, het	PVOD
PAH197	F/37	IPAH	c.594 + 1G > A, het	c.2965C > T: p.Arg989Trp, het	PVOD
PAH202	F/29	IPAH	c.986_987del: p.329_329del, hom		PVOD

*F* Female, *M* male, *het* heterozygous, *hom* homozygous

Because deletion/duplication contributed considerably to *BMPR2* pathogenic mutations in HPAH, we tried to detect CNVs in targeted NGS panel data. With the newly developed panelcn.MOPS pipeline by Povysil et al. [25], 12 *BMPR2* deletions were detected, after excluding those flagged as low quality (Table 4). To confirm the results, MLPA was performed. Almost all *BMPR2* CNVs were proved to be true, except for a slight inconsistency in patient PAH52, which panelcn.MOPS suggested only exon 11 del while MLPA revealed an exon 11–12 del in *BMPR2*, suggesting that the method implemented by panelcn.MOPS was highly effective for *BMPR2* CNV detection. Confusingly, CNVs in *ENG* and *ACVRL1* called by panelcn.MOPS were proved to be false (Additional file 1: Table S3), probably because of a high GC content (Additional file 1: Figure S3).

**Table 4:** CNVs in *BMPR2* by panelcn.MOPS and MLPA

Patient ID	Gene	Transcript	panelcn.MOPS	MLPA	Accordance
PAH22	*BMPR2*	NM_001204	Ex1 del, het	Ex1 del, het	Full
PAH36	*BMPR2*	NM_001204	Ex4 del, het	Ex4 del, het	Full
PAH44	*BMPR2*	NM_001204	The whole gene del, het	The whole gene del, het	Full
PAH52	*BMPR2*	NM_001204	Ex11 del, het	Ex11–12 del, het	Partly
PAH60	*BMPR2*	NM_001204	Ex1 del, het	Ex1 del, het	Full
PAH84	*BMPR2*	NM_001204	Ex6–7 del, het	Ex6–7 del, het	Full
PAH100	*BMPR2*	NM_001204	Ex11 del, het	Ex11 del, het	Full
PAH109	*BMPR2*	NM_001204	The whole gene del, het	The whole gene del, het	Full
PAH158	*BMPR2*	NM_001204	Ex1 del, het	Ex1 del, het	Full
PAH173	*BMPR2*	NM_001204	Ex2–3 del, het	Ex2–3 del, het	Full
PAH174	*BMPR2*	NM_001204	Ex8–9 del, het	Ex8–9 del, het	Full
PAH190	*BMPR2*	NM_001204	Ex1 del, het	Ex1 del, het	Full

*CNVs* Copy number variations, *Ex* Exon, *het* heterozygous

When trying to explore the genotype-phenotype correlation, we found that PAH patients with *BMPR2* causal and rare mutations were younger at diagnosis (27.2y vs. 31.6y, *p* = 0.0003) and had more severe pulmonary hemodynamic impairment and a worse cardiac index (2.6 L/min/m^2^ vs. 3.0 L/min/m^2^, *p* = 0.0017) (Table 5) compared with those without *BMPR2* mutations, in accord with previous reports [9]. In addition, patients with biallelic *EIF2AK4* mutations had a lower diffusion capacity for carbon monoxide of lung (DLCO) than PAH patients (Additional file 1: Table S4).

**Table 5:** Genotype-phenotype correlation between PAH patients with and without a *BMPR2* mutation

Characteristic	PAH with a *BMPR2* mutation	PAH without a *BMPR2* mutation	*P* value
Age at diagnosis, y	27.2 ± 9.9	31.6 ± 10.5	0.0003
Female sex	38 (67.9%)	99 (76.7%)	
NYHA I-II	27 (48.2%)	56 (43.4%)	
NYHA III	28 (50.0%)	67 (51.9%)	
NYHA IV	1 (1.8%)	6 (4.7%)	
Hemodynamics at diagnosis			
RAP, mmHg	5.1 ± 4.4	5.1 ± 4.2	ns
mPAP, mmHg	60.2 ± 15.3	54.9 ± 14.7	0.0321
PVR, dyn*s*cm^−5^	1386.2 ± 641.4	1042.1 ± 417.0	0.0024
PAWP, mmHg	6.9 ± 3.5	7.2 ± 3.5	ns
CI, L/min/m^2^	2.6 ± 0.9	3.0 ± 0.9	0.0017
SvO_2_, %	64.8 ± 8.1	69.4 ± 6.6	0.0005
Peak VO_2_, ml/min/Kg	11.7 ± 2.7	13.9 ± 3.6	0.0096
6-min walk distance, m	396.8 ± 106.1	427.0 ± 97.7	ns
NT-proBNP, pg/ml	1432.1 ± 1326.2	1327.4 ± 1283.9	ns

Values are mean ± SD or n (%). *NYHA* New York Heart Association functional class, *RAP* right atrial pressure, *mPAP* mean pulmonary artery pressure, *PVR* pulmonary vascular resistance, *PAWP* pulmonary artery wedge pressure, *CI* cardiac index, *SvO*_*2*_ mixed venous oxygen saturation, *Peak VO*_*2*_ peak oxygen consumption, *NT-proBNP* N-terminal pro–B-type natriuretic peptide, *ns* no significance

## Discussion

To the best of our knowledge, this was the largest reported cohort of Chinese PAH patients undergoing genetic testing, which greatly contributed to expand PAH genetic database in Asian population. In the present study, 31.3% (56/179) IPAH and 66.7% (8/12) HPAH patients were detected causative mutations. Targeted NGS panel assay is a highly effective clinical tool, which allows not only for the detection of small sequence alterations, but also for an indication of CNVs with specific computational algorithms. Our results showed that 37 *BMPR2* mutations (37/49, 75.5%) were identified in 32 IPAH and 5 HPAH by the sequencing methods and 12 *BMPR2* CNVs (12/49, 24.5%) were indicated from the panel data and finally validated by MLPA, which was distinct from Cogan’s report [8] that a higher frequency of *BMPR2* mutation was detected by deletions/duplications analysis in HPAH, compared with that by sequence analysis (48% vs. 37%). Therefore, an indication of CNVs from panel data would largely save costs in testing specified samples. Our results revealed that the newly developed and free available panelcn.MOPS pipeline [25] showed high sensitivity and specificity in exploring *BMPR2* CNVs, ranging from single exons to the whole gene, and all were confirmed by MLPA, except for a slight inconsistency in which panelcn.MOPS suggested a single exon 11 del while MLPA revealed an exon 11–12 del in *BMPR2* in patient PAH52. Conversely, CNVs detected in the genes *ENG* and *ACVRL1* were all proved to be false, which might be related to a high GC content in these two genes.

Notably, 86 IPAH patients and 4 HPAH families were not detected any suspected mutations in the 13 known genes involved in our panel, indicating that other genes potentially responsible for the disease might exist. In most recent years, more genes were discovered, such as *KLF2* [26], *SOX17* [27]*, AQP1* [27]. We would add these novel genes and other candidate genes related to PAH into our panel in the future, such as *FOXF*1 [28]*, RASA1* [29]*, TBX4* [30]*, ABCA3* [31]*, SMAD1* [15]. Furthermore, we would also dedicate in novel gene discovering through whole exome sequencing (WES) later on, especially these four HPAH families with negative results in our cohort.

PVOD, featured with extensive and diffuse constriction and/or occlusion of pulmonary veins and venules, instead of pulmonary arteriopathy, shares several clinical and hemodynamic similarities with PAH, thus often leading to a misdiagnosis of IPAH. However, PVOD patients usually have a worse prognosis, and more importantly, the administration of PAH specific therapy (vasodilators) can precipitate severe acute pulmonary edema [32]. Hence, a diagnosis of PVOD/PCH distinct from that of PAH appears extremely important and identification of biallelic *EIF2AK4* mutations makes it possible. In our study, in 6 patients clinically diagnosed with IPAH, homozygous or compound heterozygous *EIF2AK4* mutations were detected; thus, their diagnosis should be corrected to PVOD.

Our clinical data demonstrated that patients with biallelic *EIF2AK4* mutations had a lower DLCO value, suggesting a higher probability of biallelic *EIF2AK4* detection in PAH patients with DLCO values < 40% of the predicted value. However, because of our limited PVOD sample size, the cut-off value of DLCO of predicted and the corresponding detection rate required further study.

It was reported that PVOD patients carrying biallelic *EIF2AK4* mutations had a worse survival compared with IPAH patients [33] and PVOD patients without *EIF2AK4* mutations [34], and showed no response to PAH therapy [34]. Inconsistently, neither of our two PVOD patients with intravenous injection of prostacyclin analogues, PAH3 and PAH151, demonstrated pulmonary edema. Regretfully, none of these 6 patients underwent microscopic examination of lung tissue; therefore, we were unaware of the differences that might have existed between PVOD patients who showed a good response to PAH-targeted drugs and those who did not.

With the extensive development of molecular genetics in clinical services, increasing amounts of genetic data have been obtained. Researchers are dedicated to exploring genotype-phenotype correlations in causative genes and PAH phenotypes. A meta-analysis of 1550 PAH patients revealed that PAH patients with *BMPR2* mutations presented a younger age at diagnosis with more severe disease and a younger age at death or transplantation compared with non-carriers [9]. PAH patients with *ACVRL1* mutations were younger at diagnosis and had a worse prognosis than other PAH patients [35]. In accord with previous reports, our data showed that PAH patients with *BMPR2* mutations were younger at diagnosis and presented more severe pulmonary hemodynamic impairment and a worse cardiac index. Probably because of the limited sample size, we did not observe any differences between the *ACVRL1* mutation group and other PAH patients (data not shown). The genotype-phenotype correlation exploring might help predict prognoses based on genetic results, and lay foundation for further mechanism study and target therapy.

## Conclusion

Our data further expand the PAH causative gene mutation spectrum and affirm that the panelcn.MOPS pipeline is efficient for *BMPR2* CNV detection using our panel sequencing data in clinical diagnosis, which strongly suggested to perform further MLPA testing in the specific samples. This approach would effectively save costs in the routine clinical diagnosis. The target panel assay undoubtedly represents a highly valuable clinical tool and lays the foundation for further study.

## Additional file

Additional file 1: **Table S1.** PAH panel genes. **Table S2.** Variants of unknown significance (VUS) detected in the panel genes. **Table S3.** CNVs in *ENG* and *ACVRL1* by panelcn.MOPS and MLPA. **Table S4.** Genotype-phenotype correlation between biallelic *EIF2AK4* mutations carriers and other PAH patients. **Figure S1.** Molecular genetic testing schedule. **Figure S2.** 4 HPAH families without an identified causative mutation. **Figure S3.** GC content in *BMPR2, ENG* and *ACVRL1*. (DOCX 1809 kb)

## Abbreviations

ACMG: American College of Medical Genetics
ACVRL1: activin A receptor like type 1
BMPR1B: bone morphogenetic protein receptor type 1B
BMPR2: bone morphogenetic protein receptor type 2
BWA: Burrow-Wheeler Aligner
CAV1: caveolin 1
CNV: copy-number variation
DLCO: diffusing capacity of the lung for carbon monoxide
EIF2AK4: eukaryotic translation initiation factor 2 alpha kinase 4
ENG: endoglin
GDF2: growth differentiation factor 2
gnomAD: Genome Aggregation Database
HHT: hereditary hemorrhagic telangiectasias
HPAH: heritable pulmonary arterial hypertension
IPAH: idiopathic pulmonary arterial hypertension
KCNA5: potassium voltage-gated channel subfamily A member 5
KCNK3: potassium two pore domain channel subfamily K member 3
MAF: Minor allele frequency
MLPA: Multiplex ligation-dependent probe amplification
NGS: Next-generation-sequencing
NOTCH3: Notch 3
PAH: Pulmonary arterial hypertension
PAWP: Pulmonary artery wedge pressure
PCH: Pulmonary capillary hemangiomatosis
PVOD: Pulmonary veno-occlusive disease
PVR: Pulmonary vessel resistance
SMAD4: SMAD family member 4
SMAD9: SMAD family member 9
TOPBP1: DNA topoisomerase II binding protein 1
WU: Wood units
