# Supplementary material for: Genetic analyses in a cohort of 191 pulmonary arterial hypertension patients
Source: Respir Res. 2018 May 9;19:87. doi: 10.1186/s12931-018-0789-9 (PMC5944100; doi:10.1186/s12931-018-0789-9)

Table S1 PAH panel genes

| **Gene** | **Locus** | **Protein** | **Disease** | **Exons** | **Mode of inheritance** |
| --- | --- | --- | --- | --- | --- |
| *BMPR2* | 2q33.1-q33.2 | Bone morphogenetic protein receptor type-2 | PAH, PVOD | 13 | Autosomal dominant |
| *SMAD9* | 13q13.3 | Mothers against decapentaplegic homolog 9 | PAH | 9 | Autosomal dominant |
| *CAV1* | 7q31.2 | Caveolin-1 | PAH | 4 | Autosomal dominant |
| *KCNK3* | 2p23.3 | Potassium channel subfamily K member 3 | PAH | 3 | Autosomal dominant |
| *ACVRL1* | 12q13.13 | Serine/threonine-protein kinase receptor R3 | HHT | 11 | Autosomal dominant |
| *ENG* | 9q34.11 | Endoglin | HHT | 16 | Autosomal dominant |
| *EIF2AK4* | 15q15.1 | eukaryotic translation initiation factor 2 alpha kinase 4 | PVOD, PCH | 39 | Autosomal recessive |
| *BMPR1B* | 4q22.3 | Bone morphogenetic protein receptor type-1B | PAH | 19 | Autosomal dominant |
| *GDF2* | 10q11.22 | Growth/differentiation factor 2 | HHT | 2 | Autosomal dominant |
| *KCNA5* | 12p13.32 | Potassium voltage-gated channel subfamily A member 5 | PAH | 1 | Autosomal dominant |
| *NOTCH3* | 19p13.12 | Neurogenic locus notch homolog protein 3 | PAH | 33 | Autosomal dominant |
| *SMAD4* | 18q21.2 | Mothers against decapentaplegic homolog 4 | HHT | 12 | Autosomal dominant |
| *TOPBP1* | 3q22.1 | DNA topoisomerase 2-binding protein 1 | PAH | 28 | Autosomal dominant |

Table S2 Variants of unknown significance (VUS) detected in the panel genes

| Patient | Gene | Transcript | Exon/Intron | Nucleotide change | Protein change | De novo | Pathogenicity | Ref PMID | MAF |
| --- | --- | --- | --- | --- | --- | --- | --- | --- | --- |
| PAH4 | *ACVRL1* | NM_001077401 | exon5 | c.671A>G | p.Glu224Gly | Inherited | VUS | NA | NA |
| PAH6 | *ENG* | NM_000118 | exon6 | c.766C>T | p.Pro256Ser | NA | VUS | NA | 0.0001 |
| PAH7 | *EIF2AK4* | NM_001013703 | exon7 | c.755A>G | p.Gln252Arg | NA | VUS | NA | NA |
| PAH20 | *SMAD4* | NM_005359 | exon8 | c.947A>G | p.Asn316Ser | Inherited | VUS | NA | 0.0003 |
| PAH22 | *NOTCH3* | NM_000435 | exon24 | c.4348G>A | p.Ala1450Thr | NA | VUS | 22006983 | 0.0015 |
| PAH25 | *ENG* | NM_000118 | exon2 | c.214C>T | p.Pro72Ser | NA | VUS | NA | NA |
| PAH28 | *TOPBP1* | NM_007027 | exon22 | c.3687C>G | p.Ile1229Met | NA | VUS | NA | 2.69×10^-5^ |
| PAH33 | *SMAD9* | NM_005905 | exon5 | c.965G>A | p.Ser322Asn | NA | VUS | NA | NA |
| PAH36 | *BMPR2* | NM_001204 | exon6 | c.635G>A | p.Gly212Asp | NA | VUS | NA | NA |
| PAH37 | *BMPR1B* | NM_001256792 | exon2 | c.62C>T | p.Thr21Ile | NA | VUS | NA | 0.0007 |
| PAH39 | *ACVRL1* | NM_001077401 | exon6 | c.914C>A | p.Ser305Tyr | NA | VUS | NA | NA |
| PAH40 | *CAV1* | NM_001172896 | exon2 | c.185C>T | p.Thr62Ile | NA | VUS | NA | NA |
| PAH41 | *ACVRL1* | NM_001077401 | exon6 | c.847G>T | p.Gly283Cys | NA | VUS | NA | NA |
| PAH47 | *NOTCH3* | NM_000435 | exon7 | c.1159G>T | p.Ala387Ser | NA | VUS | NA | NA |
| PAH47 | *NOTCH3* | NM_000435 | exon20 | c.3299G>A | p.Arg1100His | NA | VUS | NA | 0.0016 |
| PAH48 | *EIF2AK4* | NM_001013703 | exon36 | c.4724T>C | p.Leu1575Pro | NA | VUS | NA | NA |
| PAH56 | *KCNA5* | NM_002234 | exon1 | c.1570C>T | p.His524Tyr | NA | VUS | NA | NA |
| PAH57 | *BMPR2* | NM_001204 | exon12 | c.2742T>G | p.Asp914Glu | NA | VUS | NA | 5.8×10^-5^ |
| PAH58 | *NOTCH3* | NM_000435 | exon33 | c.6449G>A | p.Arg2150His | NA | VUS | NA | 4.22×10^-5^ |
| PAH61 | *BMPR2* | NM_001204 | exon9 | c.1274C>T | p.Pro425Leu | NA | VUS | NA | NA |
| PAH61 | *TOPBP1* | NM_007027 | exon14 | c.2287G>A | p.Ala763Thr | NA | VUS | NA | 0.0002 |
| PAH64 | *BMPR2* | NM_001204 | exon11 | c.1486T>C | p.Cys496Arg | NA | VUS | NA | NA |
| PAH65 | *ACVRL1* | NM_001077401 | exon9 | c.1436G>A | p.Arg479Gln | NA | VUS | NA | NA |
| PAH65 | *EIF2AK4* | NM_001013703 | exon9 | c.1256A>G | p.Asn419Ser | NA | VUS | NA | 0.0002 |
| PAH67 | *EIF2AK4* | NM_001013703 | exon6 | c.719G>A | p.Arg240Gln | Inherited | VUS | NA | 0.0001 |
| PAH69 | *BMPR2* | NM_001204 | exon7 | c.967G>A | p.Asp323Asn | NA | VUS | NA | NA |
| PAH71 | *SMAD9* | NM_001127217 | exon2 | c.194C>T | p.Pro65Leu | NA | VUS | NA | 5.8×10^-5^ |
| PAH73 | *GDF2* | NM_016204 | exon2 | c.1283G>T | p.Cys428Phe | Inherited | VUS | NA | NA |
| PAH74 | *NOTCH3* | NM_000435 | exon33 | c.6100C>G | p.Pro2034Ala | NA | VUS | NA | 6.22×10^-5^ |
| PAH85 | *ACVRL1* | NM_001077401 | exon7 | c.1108G>A | p.Val370Met | NA | VUS | NA | NA |
| PAH90 | *KCNK3* | NM_002246 | exon2 | c.520G>A | p.Gly174Ser | NA | VUS | NA | NA |
| PAH91 | *SMAD9* | NM_001127217 | exon2 | c.347C>T | p.Pro116Leu | NA | VUS | NA | 5.96×10^-5^ |
| PAH97 | *NOTCH3* | NM_000435 | exon25 | c.4567G>C | p.Glu1523Gln | NA | VUS | NA | NA |
| PAH105 | *KCNA5* | NM_002234 | exon1 | c.68G>A | p.Arg23Gln | NA | VUS | NA | 0 |
| PAH105 | *NOTCH3* | NM_000435 | exon31 | c.5764G>T | p.Val1922Leu | NA | VUS | NA | 0.0005 |
| PAH111 | *TOPBP1* | NM_007027 | exon12 | c.1881T>G | p.Asp627Glu | NA | VUS | NA | NA |
| PAH121 | *EIF2AK4* | NM_001013703 | exon6 | c.626A>G | p.Gln209Arg | NA | VUS | NA | 0.0008 |
| PAH122 | *BMPR1B* | NM_001256792 | exon9 | c.1239A>T | p.Arg413Ser | NA | VUS | NA | 0.0017 |
| PAH123 | *BMPR2* | NM_001204 | intron3 | IVS3+4A>G |  | NA | VUS | NA | NA |
| PAH137 | *ACVRL1* | NM_001077401 | exon7 | c.1244A>G | p.Asn415Ser | NA | VUS | NA | NA |
| PAH140 | *ACVRL1* | NM_001077401 | exon7 | c.1202T>C | p.Phe401Ser | NA | VUS | NA | NA |
| PAH141 | *NOTCH3* | NM_000435 | exon4 | c.668C>T | p.Ala223Val | NA | VUS | NA | 0.0001 |
| PAH147 | *TOPBP1* | NM_007027 | exon14 | c.2354G>A | p.Arg785His | NA | VUS | NA | 6.54×10^-5^ |
| PAH148 | *GDF2* | NM_016204 | exon1 | c.307A>T,hom | p.Thr103Ser | NA | VUS | NA | NA |
| PAH152 | *EIF2AK4* | NM_001013703 | exon12 | c.1955A>G | p.Tyr652Cys | NA | VUS | NA | 9.15×10^-6^ |
| PAH152 | *NOTCH3* | NM_000435 | exon4 | c.472G>A | p.Asp158Asn | NA | VUS | NA | 0.0006 |
| PAH156 | *ACVRL1* | NM_001077401 | exon5 | c.631G>A | p.Gly211Ser | Inherited | VUS | NA | NA |
| PAH165 | *ENG* | NM_000118 | exon2 | c.214C>T | p.Pro72Ser | NA | VUS | NA | NA |
| PAH180 | *BMPR2* | NM_001204 | exon12 | c.2742T>G | p.Asp914Glu | Inherited | VUS | NA | 5.8×10^-5^ |
| PAH180 | *NOTCH3* | NM_000435 | exon33 | c.6103G>T | p.Gly2035Cys | Inherited | VUS | NA | NA |
| PAH181 | *ACVRL1* | NM_001077401 | exon1 | c.23A>C | p.Lys8Thr | NA | VUS | NA | 5.8×10^-5^ |
| PAH183 | *EIF2AK4* | NM_001013703 | exon26 | c.3589A>G | p.Ser1197Gly | NA | VUS | NA | 0.0002 |
| PAH185 | *KCNK3* | NM_002246 | exon2 | c.544G>A | p.Glu182Lys | NA | VUS | 23883380 | NA |
| PAH185 | *NOTCH3* | NM_000435 | exon24 | c.4039G>C | p.Gly1347Arg | NA | VUS | NA | NA |
| PAH191 | *EIF2AK4* | NM_001013703 | exon29 | c.3979A>G | p.Ile1327Val | NA | VUS | NA | 0.0005 |
| PAH193 | *BMPR2* | NM_001204 | intron1 | IVS1+5G>T |  | NA | VUS | NA | NA |
| PAH200 | *NOTCH3* | NM_000435 | exon25 | c.4429G>T | p.Asp1477Tyr | NA | VUS | NA | NA |
| PAH203 | *SMAD9* | NM_001127217 | exon2 | c.173C>T | p.Ala58Val | Inherited | VUS | NA | NA |
| PAH204 | *ACVRL1* | NM_001077401 | exon7 | c.1232G>A | p.Arg411Gln | NA | VUS | 8640225 | 6.55×10^-5^ |
| PAH204 | *EIF2AK4* | NM_001013703 | exon9 | c.1388G>A | p.Arg463Gln | NA | VUS | NA | 8.95×10^-6^ |
| PAH205 | *GDF2* | NM_016204 | exon2 | c.726_727del | p.242_243del | Inherited | VUS | NA | NA |

NA, not available; VUS, variants of unknown significance; ref PMID, reference PubMed unique identifier; MAF, minor allele frequency in the Genome Aggregation Database (gnomAD).

Table S3 CNVs in *ENG* and *ACVRL1* by panelcn.MOPS and MLPA

| Patient ID | Gene | Transcript | panelcn.MOPS | MLPA | Accordance |
| --- | --- | --- | --- | --- | --- |
| PAH72 | *ENG* | NM_001114753 | Ex1 del, het | Negative | No |
| PAH80 | *ENG* | NM_001114753 | Ex7 del, het | Negative | No |
| PAH115 | *ENG* | NM_001114753 | Ex7 del, het | --- | NA |
| PAH192 | *ENG* | NM_001114753 | Ex9 del, het | --- | NA |
| PAH71 | *ACVRL1* | NM_000020 | Ex1 del, het | --- | NA |
| PAH72 | *ACVRL1* | NM_000020 | Ex1 del, het | Negative | No |
| PAH186 | *ACVRL1* | NM_000020 | Ex1 del, het | Negative | No |
| PAH203 | *ACVRL1* | NM_000020 | Ex1 del, het | --- | NA |

NA, not available; --- indicates that the sample had not been performed MLPA.

Table S4 Genotype-phenotype correlation between biallelic *EIF2AK4* mutations carriers and other PAH patients

| **Characteristic** | **biallelic *EIF2AK4* mutations carriers(n=6)** | **other PAH patients (n=83)** | **P value** |
| --- | --- | --- | --- |
| Age at diagnosis, y | 37 ± 16.1 | 31.6 ± 10.5 | ns |
| Female sex | 4 (67%) | 66 (79.5%) |  |
| NYHA I-II | 4 (66.7%) | 43 (51.8%) |  |
| NYHA III | 2 (33.3%) | 38 (45.8%) |  |
| NYHA IIIIV | 0 (-) | 2 (2.4%) |  |
| Hemodynamics at diagnosis |  |  |  |
| DLCO(%pred) | 30.7 ± 5.13 | 67.4±11.17 | <0.0001 |
| RAP, mmHg | 7.5 ± 0.7 | 5.0 ± 3.9 | ns |
| mPAP, mmHg | 52.3 ± 5.1 | 56.1 ± 16.2 | ns |
| PVR, dyn*s*cm^-5^ | 938.4 ±253.2 | 1101.3 ± 474.6 | ns |
| PAWP, mmHg | 9 ± 1 | 6.5 ± 3.5 | ns |
| CI, L/min/m^2^ | 3.9 ± 1.8 | 3.0 ± 0.9 | ns |
| SvO_2_, % | 68.9 ± 8.0 | 68.4 ± 7.2 | ns |
| Peak VO_2_, ml/min/Kg | 10.7 ± 2.4 | 13.2 ± 3.6 | ns |
| 6-min walk distance, m | 359 ± 151.5 | 419.9 ± 100 | ns |
| NT-proBNP, pg/ml | 798.6 ± 897.2 | 1154.0 ± 1195.7 | ns |

Values are mean ± SD or n (%). NYHA, New York Heart Association functional class; DLCO, carbon monoxide of lung; %pred, percentage of predicted value; RAP, right atrial pressure; mPAP, mean pulmonary artery pressure; PVR, pulmonary vascular resistance; PAWP, pulmonary artery wedge pressure; CI, cardiac index; SvO_2_, mixed venous oxygen saturation; Peak VO_2_, peak oxygen consumption; NT-proBNP, N-terminal pro–B-type natriuretic peptide; ns, no significance.

Figure S1 Molecular genetic testing schedule


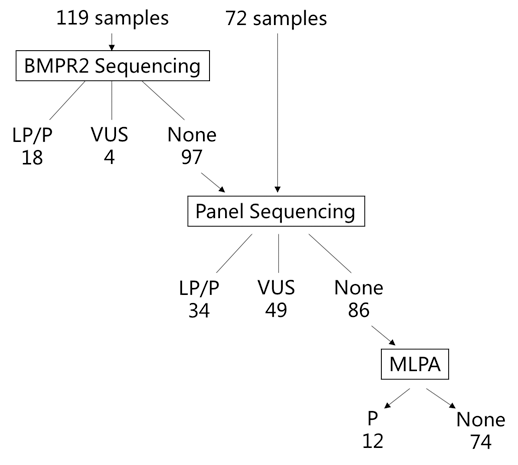


Note: LP, likely pathogenic; P, pathogenic; VUS, variants of unknown significance.

Figure S2 4 HPAH families without an identified causative mutation


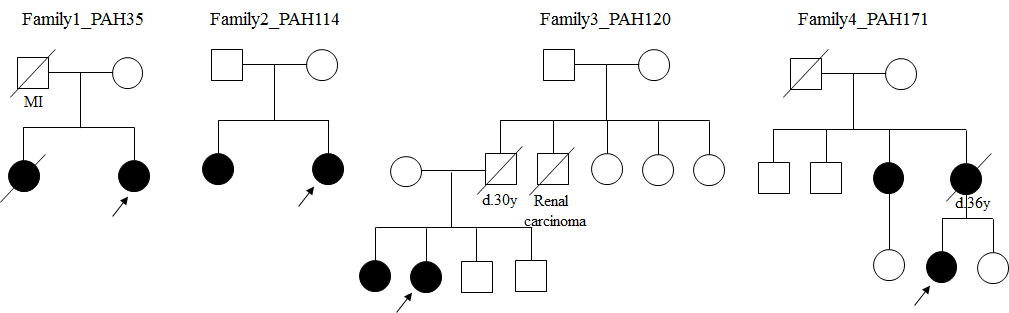


Figure S3 GC content in *BMPR2*, *ENG* and *ACVRL1*


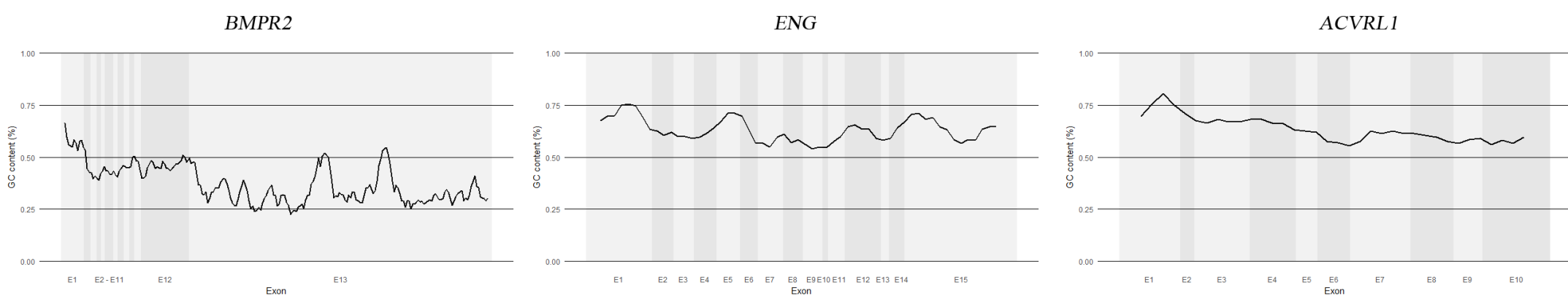

Supplement: Supplementary file 1 — Table S1. PAH panel genes. Table S2. Variants of unknown significance (VUS) detected in the panel genes. Table S3. CNVs in ENG and ACVRL1 by panelcn.MOPS and MLPA. Table S4. Genotype-phenotype correlation between biallelic EIF2AK4 mutations carriers and other PAH patients. Figure S1. Molecular genetic testing schedule. Figure S2. 4 HPAH families without an identified causative mutation. Figure S3. GC content in BMPR2, ENG and ACVRL1. (DOCX 1809 kb) [file 12931_2018_789_MOESM1_ESM.docx]
